# *Fusobacterium nucleatum* Promotes Metastasis in Colorectal Cancer by Activating Autophagy Signaling via the Upregulation of CARD3 Expression

**DOI:** 10.7150/thno.38870

**Published:** 2020-01-01

**Authors:** Yongyu Chen, Yan Chen, Jixiang Zhang, Pan Cao, Wenhao Su, Yunchao Deng, Na Zhan, Xiangsheng Fu, Yun Huang, Weiguo Dong

**Affiliations:** 1 Department of Gastroenterology, Renmin Hospital of Wuhan University, Wuhan, Hubei Province, China.; 2 Key Laboratory of Hubei Province for Digestive System Disease, Wuhan, Hubei Province, China.; 3 Central Laboratory, Renmin Hospital of Wuhan University, Wuhan, Hubei Province, China.; 4 Department of Gastroenterology, The Affiliated Hospitalof North Sichuan Medical College, Road Wenhua 63#, Region Shunqing, Nanchong City 637000, China.; 5 Center for Epigenetics & Disease Prevention, Institute of Biosciences and Technology, Texas A&M University, Houston, TX77030, USA.

**Keywords:** microbe, gene regulation, gene targeting, colorectal cancer

## Abstract

**Aims**: We aimed to measure the abundance of *Fusobacterium nucleatum* (*F. nucleatum*) in colorectal cancer (CRC) tissues from patients and to uncover the function of this bacterium in colorectal tumor metastasis.

**Methods**: We collected metastatic and non-metastatic CRC tissues to analyze *F. nucleatum* abundance. Cells were incubated with *F. nucleatum* or chloroquine (CQ) or were transfected with CARD3-targeting siRNA; the expression of mRNAs and proteins was then measured. CRC cells stably transfected with shRNA-luc were mixed with *F. nucleatum* and intravenously injected into BALB/cJ mice. APC^Min/+^, CARD3^-/-^and CARD3^wt^ C57BL mice were given *F. nucleatum*; some mice were given azoxymethane (AOM) and dextran sodium sulfate (DSS).

**Results**: *F. nucleatum* was abundant in CRC tissues from patients with metastasis. *F. nucleatum* infection increased CRC cell motility and upregulated the expression of CARD3, LC3-II, Beclin1 and Vimentin, and downregulated the expression of E-cadherin and P62 in CRC cells. These effects were attenuated by treatment with CQ, siCARD3 or both. APC^Min/+^ mice gavaged with *F. nucleatum* developed more aggressive tumors than control mice. After AOM/DSS administration, the colorectums of CARD3^-/-^ mice had fewer tumors than those of control mice. Tumors from CARD3^-/-^ mice had lower levels of LC3-II and Beclin1 and higher levels of P62 than those from control mice. BALB/cJ mice injected with both CT26-luc cells and *F. nucleatum* formed more metastases than control mice. CQ treatment, CARD3 knockdown or both reduced the ability of CT26-luc cells to form metastases *in vivo*.

**Conclusions**: *F. nucleatum* is enriched in CRC tissues from patients with metastasis. *F. nucleatum* orchestrates CARD3 and autophagy to control CRC metastasis. Measuring and targeting *F. nucleatum* and its associated pathways will yield approaches for the prevention and treatment of CRC metastasis.

## Introduction

Colorectal cancer (CRC) is the third-leading cause of cancer-related death 1. Approximately 50%-60% of CRC patients develop metastasis, which is the main cause of mortality in patients 2. Despite various clinical advances in chemotherapy and targeted agents for patients with metastatic CRC, the 5-year survival rate remains unsatisfactory 3,4. Therapeutic failure is often associated with metastatic spread 5,6. Thus, it is important to elucidate the mechanism underlying metastasis in CRC patients.

Metastasis results from complex interplay between the environment and gene regulation. The microbiota is associated with CRC initiation and progression via intestinal inflammation and tumor-related signaling pathways 7,8. Some groups have shown that *F. nucleatum* abundance is gradually increased during colorectal carcinogenesis 9,10. Moreover, the abundance of *F. nucleatum* in CRC tissues is associated with cancer initiation, proliferation, invasion, recurrence, chemoresistance and reduced survival 11-13. *F. nucleatum* attaches to E-cadherin via the fusobacterial adhesin FadA and activates the TLR4/MYD88, nuclear factor-kappa B (NF-κB), autophagy or Wnt pathway to promote cancer initiation, proliferation, invasion, recurrence or chemoresistance 11,13,14. In addition, *Fusobacterium* can be detected in liver metastases by fluorescence *in situ* hybridization (FISH), suggesting that *Fusobacterium* may migrate with CRC cells to the metastatic site 15. However, the potential effects and mechanisms of *F. nucleatum* in metastasis have not been examined.

A recent study showed that *F. nucleatum* can activate the autophagy pathway in CRC 13. Autophagy, a precisely regulated lysosomal housekeeping process, has been demonstrated to participate in cancer metastasis 16,17. Autophagy inhibition reduces the migration and invasion of tumor cells *in vitro* and attenuates metastasis *in vivo* 18. The role of autophagy in metastasis is multifaceted and depends on the tumor microenvironment (TME) and cell types. Specifically, autophagy regulates several mechanisms contributing to metastasis, including integrin signaling and trafficking, cytoskeletal remodeling, and epithelial-mesenchymal transition (EMT) signaling 16,19. However, the exact molecular mechanism by which autophagy is involved in CRC metastasis is still unclear.

Caspase activation and recruitment domain 3 (CARD3, RIP2), which is known for its role in inflammation and immunity, is a serine/threonine/tyrosine kinase with a carboxy-terminal caspase activation and recruitment domain (CARD) 20. Activation of Nod-like receptors (NLRs; Nod1 and Nod2) initiates a proinflammatory response dependent mainly on the recruitment of the adaptor protein CARD3 21. Previous studies have found that NOD1 detects gram-negative bacterial peptidoglycan (PG) within early endosomes, thereby promoting RIP2-dependent autophagy and inflammatory signaling in response to bacterial infection 22,23. Moreover, CARD3 is a prometastatic kinase in patients with advanced breast cancer 24. However, the potential effects and mechanisms of CARD3 in *F. nucleatum*-associated CRC have not been examined.

Here, we tested whether and how *F. nucleatum* affects metastasis in CRC patients. We found that *F. nucleatum* abundance is increased in metastatic CRC compared with non-metastatic CRC. We demonstrated that *F. nucleatum* plays a critical role in mediating CRC metastasis via upregulation of CARD3 and activation of the autophagy pathway.

## Methods

### Human specimens

The institutional review board of Renmin Hospital of Wuhan University approved the use of human samples for this study (approval number: 2017K-C055). All human samples were obtained with informed consent from patients with CRC who did not receive preoperative local or systemic anticancer treatment and did not use antibiotic treatment for at least 3 months. The tumor stage was classified according to the 8^th^ edition of the UICC/AJCC TNM staging system for CRC. The samples from non-metastatic (AJCC Ⅰ-Ⅱ) and metastatic (AJCC Ⅲ-Ⅳ) CRC tissues and from adjacent normal mucosa (para-tumor tissue at least 5 cm from the margin of the tumor) used for high-throughput sequencing and real-time PCR were obtained from patients undergoing surgery at Renmin Hospital of Wuhan University. After collection, all tissue samples were immediately frozen in liquid nitrogen and stored at -80 °C until use. The frozen and formalin-fixed paraffin-embedded blocks (62 metastatic CRC and 32 non-metastatic CRC and matched lymph node samples) used for FISH and immunohistochemical staining were collected from the pathology department of the same hospital. Clinicopathological data for each patient were obtained from hospital records.

### Bacterial strains and growth conditions

*F. nucleatum* strain (F01) and *F. nucleatum* strain (ATCC10953) were kindly supplied by Dr. Xiangsheng Fu of The Affiliated Hospital of North Sichuan Medical College. *F. nucleatum* strains were incubated for 3-4 days in FAB under anaerobic conditions at 37 °C. *E. coli* strain (Tiangen, China) were cultured in Luria-Bertani (LB) medium for 12-16 h at 37 °C under shake cultivation at 200-220 rpm/min.

### Cell lines

The human CRC cell lines SW480 and HCT116 and the mouse CRC cell line CT26 (ATCC) were cultured in high-glucose DMEM (Gibco, Carlsbad, CA) supplemented with 10% fetal bovine serum (FBS, Gibco) at 37 °C in a humidified 5% CO_2_ atmosphere.

### Mice

Five to six-week-old male C57BL/6J-adenomatous polyposis coli mice (APC^Min/+^), 5- to 6-week-old male C57BL/6J wild-type (CARD3^wt^) mice and 6- to 8-week-old female BALB/cJ mice were obtained from Nanjing Biomedical Research Institute of Nanjing University (NBRI). Five- to six-week-old male C57BL/6J CARD3 knockout (KO, CARD3^-/-^) mice were kindly provided by Dr. Richard Flavell (Howard Hughes Medical Institute, Yale University, New Haven, CT). All animal protocols were approved by the Animal Care and Use Committee of Renmin Hospital of Wuhan University, China (approval number: 20181001). All mice were housed and reared under SPF barrier conditions and fed autoclaved food and water. Before intragastric administration of bacteria, APC^Min/+^, CARD3^-/-^ or CARD3^wt^ mice were given streptomycin (2 mg/ml) in the drinking water for 3 days. PBS-resuspended *F. nucleatum* (F01, 10^9^ CFU/ml) or PBS was administered to mice by gavage daily. In the APC^Min/+^ mouse model, bacteria, PBS or CQ (50 mg/kg, intraperitoneal injection, every 3 days) was administered for a period of 8 weeks. In the colitis-associated cancer model, CARD3^-/-^ or CARD3^wt^ mice first received one intraperitoneal injection of the carcinogen azoxymethane (AOM) at a dose of 12.5 mg/kg body weight; were then given three sets of doses of 3% dextran sodium sulfate (DSS) for 5 days beginning on days 5, 26, and 47; and were given regular drinking water thereafter. During the DSS intervention, the bacterial feeding experiments were suspended. For assays to measure the formation of lung metastases, 5×10^6^ to 1×10^7^ *F. nucleatum* (F01) were washed twice with PBS. CT26 cells stably transfected with the luciferase-labeled CARD3 gene (shCARD3-luc) or control (NC-luc) were mixed with 10^6^ to 1×10^7^ *F. nucleatum* (F01) or PBS and injected into BALB/cJ mice via the tail vein. The animals were then intravenously administered CQ (30 mg/kg body weight) every three days. The development of metastases was monitored by BLI (Xenogen IVIS 200 Imaging System). After 24 days, the number and size of lung, liver and colon metastases were assessed. All mice were anesthetized with pentobarbital sodium (40 mg/kg) and sacrificed for analysis. The tumors were counted, and the tumor sizes (diameter) were quantified as < 1 mm, 2-3 mm, 4-5 mm, or > 5 mm.

### Luciferase imaging

D-luciferin (The *In vivo* Imaging Community) at a dose of 1.5 mg/10 g body weight was injected intraperitoneally into mice, and 10 min later, luciferase imaging (Xenogen IVIS-200) was performed.

### Cell transfection

siRNAs targeting the human CARD3 gene (siCARD3) and non-targeting siRNAs (control siRNAs) were purchased from Guangzhou RiboBio Co., Ltd. (Guangzhou, China). The specific siRNAs targeting CARD3 were as follows: si-h-CARD3 001, 5'-GAGAACATTTGAAGAGATA-3'; si-h-CARD3 002, 5'-CAATATGACTCCTCCTTTA-3'; si-h-CARD3 003, 5'-GAAAGAGGACTATGAACTT-3'. A non-targeting sequence (5'-TTCTCCGAACGTGTCACGT-3') was set as the negative control (NC). Lipid-based transfections were performed with Lipofectamine 6000 (Beyotime, China) according to the manufacturer's protocol. Cells were incubated with the siRNA complex for 72 h, and protein was extracted for assessment of transfection efficiency by Western blotting (Figure S1J). Cells transfected with si-h-CARD3 002 were collected for further studies.

### Knockdown of CARD3 with shRNA

Lentivirus harboring a plasmid containing short hairpin RNA (shRNA) against CARD3 was purchased from GeneChem (Shanghai, China). The target sequence of the shRNA against the mouse CARD3 gene was 5′‐ACGAGAAGCCGAAAUAUUA‐3′. A vector containing non‐silencing short hairpin RNA was used as the control. Lentiviral infection was performed according to the manufacturer's protocol.

### Cell migration and invasion assays

Cell lines were transfected with the indicated siRNAs for 48 h before treatment with CQ (20 μM, Sigma-Aldrich). After pretreatment with CQ for 3 h, cell lines were incubated with *F. nucleatum* (F01) at a multiplicity of infection (MOI) of 100:1 for another 24 h. Migration or invasion assays were performed as previously described 25.

### High-throughput sequencing

DNA from frozen biopsies or total RNA from HCT116 cells was extracted via a TRIzol-based technique according to the manufacturer's protocol. The quantity and purity of the total isolated DNA or RNA were determined using an Eppendorf BioPhotometer D30. DNA or RNA was sent to Adaptive Biotechnology (Huada, China) for sequencing. The quality of each sample was analyzed on an Agilent 2100 Bioanalyzer. An Illumina HiSeq 2500 was used for 250 bp paired-end sequencing of DNA samples. The splicing and cleaning of the chimera were followed by the use of FLASH version 1.2.11 26 and UCHIME version 4.2.40 27, respectively. The sequences were clustered into operational taxonomic units (OTUs) at a sequence similarity cutoff of 97% using UPARSE version 7.0.1090 28, and all OTUs were assigned to the database Greengene_2013_5_99 by the QIIME software pipeline 29. mRNA was enriched with poly-A selection, and 50 base single-end RNA-seq was completed on the BGISEQ-500 platform. Sequencing reads were aligned to the human genome (hg19). Raw reads were filtered using SOAP and SOAPnuke 30 (parameters set: -l15 -q0.2 -n 0.05), and clean reads were mapped to the transcriptome in the RefSeq database using Bowtie2 31 (parameters set: -q -phred64 -sensitive -dpad0 -gbar 99999999 -mp1, 1 -np1 -score-minL, 0, -0.1 -p16 -k200). Gene expression was counted by RSEM (default) 32 and normalized as FPKM (transcripts per kilobase of exon model per million mapped reads). We used PossionDis (fold change ≥ 2.00 and FDR ≤ 0.001) to evaluate differential expression. The DNA sequence data have been deposited in the NCBI Sequence Read Archive (SRA) database (https://dataview.ncbi.nlm.nih.gov/object/PRJNA531761?reviewer=4n4d3107unupa6bmr0ojnhlr0o). The RNA sequencing data have been deposited in the NCBI SRA database (https://www.ncbi.nlm.nih.gov/sra/?term=PRJNA543426).

### DNA extraction, RNA extraction and Real-Time PCR

Genomic DNA was extracted using a TIANamp Bacteria DNA Kit (Tiangen, China) according to the manufacturer's protocol. RNA extraction and quantitative real-time PCR were performed as previously described 33. The primers are shown in Supplementary Table 1.

### Electron microscopy

HCT116 cells were treated as indicated and fixed with 2.5% glutaraldehyde. Samples were fixed with 1% osmium tetroxide, and subsequently dehydrated in an increasing concentration gradient of ethanol and propylene oxide. Then, samples were embedded, cut into 70 nm sections, and stained with 3% uranyl acetate and lead citrate. Images were acquired using a JEM-1230 electron microscope (JEOL, Tokyo, Japan).

### Fluorescence *in situ* hybridization (FISH)

Five-micrometer-thick sections were prepared and hybridized as described in our previous study 34. The sequence of the “universal bacterial” probe (EUB338; Cy3-labeled) was 5'-GCT GCC TCC CGT AGG AGT-3'. The sequence of the *F. nucleatum* -targeted probe (FUS664; FITC-labeled) was 5'-CTT GTA GTT CCG C(C/T) TAC CTC-3'. Slides were examined using a microscope (BX53F; Olympus, Tokyo, Japan). Five random 200× magnification fields per sample were evaluated by three observers blinded to the experimental protocol, and the average number of bacteria per field was calculated. We defined a low or high abundance of *F. nucleatum* as an average of < 20 or > 20 visualized FUS664 probes per field, respectively. The presence of other bacteria was considered positive for samples exhibiting > 5 bacteria per field with EUB338 probe, but negative with FUS664 probe.

### Immunofluorescence and confocal microscopy

Immunofluorescence staining was performed as previously described 35. LC3 puncta were examined with a confocal microscope (Olympus FV1200) fitted with a 100× oil immersion objective.

### Immunohistochemistry (IHC) and Western blotting (WB)

For IHC, an UltraSensitive^TM^ SP (Mouse/Rabbit) IHC Kit (Maxim; Fuzhou, China) was used following the manufacturer's instructions. The immunoreactive score ranges from 0 to 12 and is the product of multiplication between the positive-cell proportion score (0-4) and the staining intensity score (0-3). WB was conducted as previously described 36. Primary antibodies against the following targets were used: CARD3 (CST), LC3-Ⅱ (CST), Beclin1 (CST), P62 (Sigma), E-cadherin (CST), Vimentin (Proteintech) and GAPDH (Bioworld).

### Computational analysis and LIR motif prediction

A dataset (GSE21510) consisting of 148 tumors from CRC patients was downloaded from the GEO database. According to the median value (6.45) of CARD3 RNA expression, the 148 tumors were divided into the high (> 6.45; n = 88) and low (< 6.45; n = 60) CARD3 expression groups. GSEA was performed as previously described 37. The LIR motif in human and mouse CARD3 was predicted using the iLIR web server as previously described 38.

### Statistical analyses

Differences in quantitative data between two groups were compared using paired or unpaired Student's *t*-tests, Mann-Whitney U-tests, or Dunnett's *t*-tests as appropriate. The relationships between the abundance of *F. nucleatum* and mRNA expression were analyzed by linear regression. Associations between *F. nucleatum* abundance and patient characteristics were determined using Pearson's chi-squared test or Fisher's exact test, as appropriate. The median value was selected as the cutoff point. All *P* values were two-tailed, and differences with a *P* value of less than 0.05 were considered significant differences (*P < 0.05, ** *P* < 0.01, and *** *P* < 0.001). All statistical analyses were conducted using GraphPad Prism 6 software (GraphPad software, Inc., San Diego, California, USA) and SPSS Statistics 20.0 software (IBM, Inc., Chicago, Illinois, USA).

## Results

### *F. nucleatum* is Associated with Colorectal Cancer Metastasis

**Figure 1:**
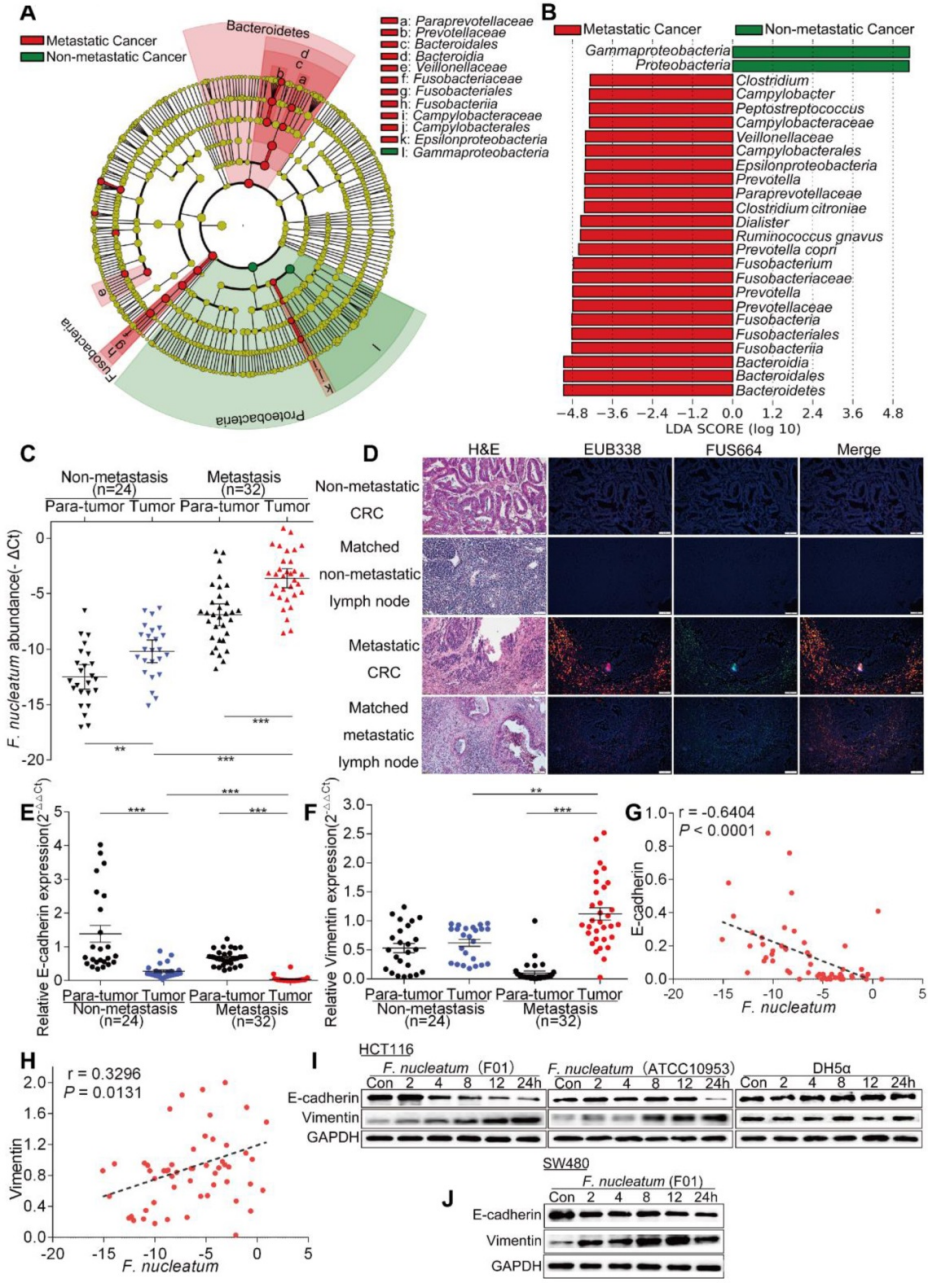
***F. nucleatum* is Associated with Colorectal Cancer Metastasis.** (A) A cladogram representation of by 16S rDNA sequencing data from CRC patients with (n = 9) and without (n = 7) metastasis. Taxa enriched in patients with metastasis (red) and without metastasis (green). The brightness of each dot is proportional to its effect size. (B) Linear discriminant analysis (LDA) coupled with effect size measurements identified the significantly differential abundances in the data referenced in A. Only taxa with values greater than the LDA threshold of 3.6 are shown. (C) Statistical analysis of the abundance of *F. nucleatum* in CRC patients with or without metastasis (**P < 0.01, and ***P < 0.001; nonparametric Mann-Whitney test). (D) Representative images of FISH to assess the amount of *F. nucleatum* in metastatic CRC tissues (n = 62), non-metastatic CRC tissues (n = 32) and matched lymph nodes. EUB338 (red) is a Cy3-conjugated “universal bacterial” oligonucleotide probe; FUS664 (green) is a FITC-conjugated *F. nucleatum* oligonucleotide probe. 200× magnification. (E-H) Statistical analysis of the mRNA expression of E-cadherin and Vimentin in CRC patients with or without metastasis (E-F; *P < 0.05, **P < 0.01, and ***P < 0.001; nonparametric Mann-Whitney test; the error bars indicate the SDs). Correlation analysis of the abundance of *F. nucleatum* and the expression levels of E-cadherin and Vimentin in cancer tissues (G-H; two-tailed, nonparametric Spearman correlation). (I-J) Western blot analysis was performed with CRC cells cocultured with *F. nucleatum* (F01), *F. nucleatum* (ATCC10951), *E. coli* or PBS (control).

To examine the potential relationship between gut microbiota alterations and CRC metastasis, we compared the sequencing data generated with the HiSeq 2500 platform from 9 metastatic CRC tissues and 7 non-metastatic CRC tissues. Using the LEfSe algorithm to define the potential differential bacterial patterns between metastatic and non-metastatic CRC tissues from patients, we observed that *Fusobacteriaceae*, *Prevotellaceae* and *Veillonellaceae* may play important roles in metastatic colorectal cancer patients compared with non-metastatic colorectal cancer patients (*P* < 0.01; Figure 1A). We found that *Fusobacterium*, *Prevotella* and *Dialister* were enriched in metastatic CRC tissues (*P* < 0.01; Figures 1B, S1A). Given the role of *Fusobacterium*, especially *F. nucleatum*, in CRC, we further studied* F. nucleatum*. Consistent with the sequencing data, the real-time PCR results showed that the abundance of *F. nucleatum* in CRC tissues was higher in metastatic patients than in non-metastatic patients (*P* < 0.01; Figure 1C). Furthermore, *F. nucleatum* was more highly enriched in CRC tissues than in adjacent normal tissues in both the metastatic and non-metastatic groups (*P* < 0.01; Figure 1C). This finding suggests that *F. nucleatum* may play a role in CRC metastasis.

We further examined *F. nucleatum* abundance in 62 metastatic CRC tissues, 32 non-metastatic CRC tissues and matched lymph nodes using FISH. *F. nucleatum* was detected in 75.81% of the metastatic CRC tissues, a higher percentage than in non-metastatic CRC tissues (43.75%; *P* = 0.01). Moreover, *F. nucleatum* was detected in a higher percentage of matched lymph nodes with metastases (62.90%) than lymph nodes with no metastases (34.37%;* P* = 0.003; Figure 1D). This result shows that *F. nucleatum* is present in metastases. We then evaluated the relationship between the amount of *F. nucleatum* and different clinicopathological features as shown in Table 1. *F. nucleatum* abundance was positively associated with the American Joint Committee on Cancer (AJCC) stage, location, depth of invasion, lymph node metastasis and distant metastasis (*P* < 0.05). Thus, these data (Figure 1D, Table 1) not only confirmed our observation in Figure 1A-B, but also defined the potential value of *F. nucleatum* abundance in predicting CRC metastasis.

Tumor metastasis is facilitated by EMT. To further validate whether *F. nucleatum* promotes cancer metastasis, we used real-time PCR to analyze the expression of EMT markers in 24 non-metastatic CRC tissues and 32 metastatic CRC tissues from patients. The expression of the epithelial marker E-cadherin in CRC tissues was lower in metastatic patients than in non-metastatic patients, and the mesenchymal marker Vimentin was upregulated in metastatic CRC tissues (*P* < 0.05; Figure 1E-F). Combined with the data in Figure 1C, these results indicated that the abundance of *F. nucleatum* was negatively associated with E-cadherin expression (*P* < 0.0001; Figure 1G) and positively associated with Vimentin expression (*P* = 0.0131; Figure 1H) in CRC tissues. We further investigated whether *F. nucleatum* promotes the metastatic potential of CRC cells by regulating EMT. HCT116 cells and SW480 cells were cocultured with *F. nucleatum* (F01, accession number: SUB1766768 Seq01 KX692281) 36 obtained from the tissues of CRC patients, and *F. nucleatum* (ATCC10953) from the oral cavity and *Escherichia coli* (*E. coli*) DH5a were used as controls. Western blot analysis showed that *F. nucleatum* (F01 and ATCC10953) exposure decreased E-cadherin expression and increased Vimentin expression in HCT116 cells (Figure 1I) and SW480 cells (Figure 1J) in a time-dependent manner (*P* < 0.05). These effects were not found in CRC cells cocultured with *E. coli* DH5a. These results indicate that *F. nucleatum* possibly promotes metastasis via EMT.

### *F. nucleatum* Promotes Cancer-Related Autophagy Activation

**Figure 2:**
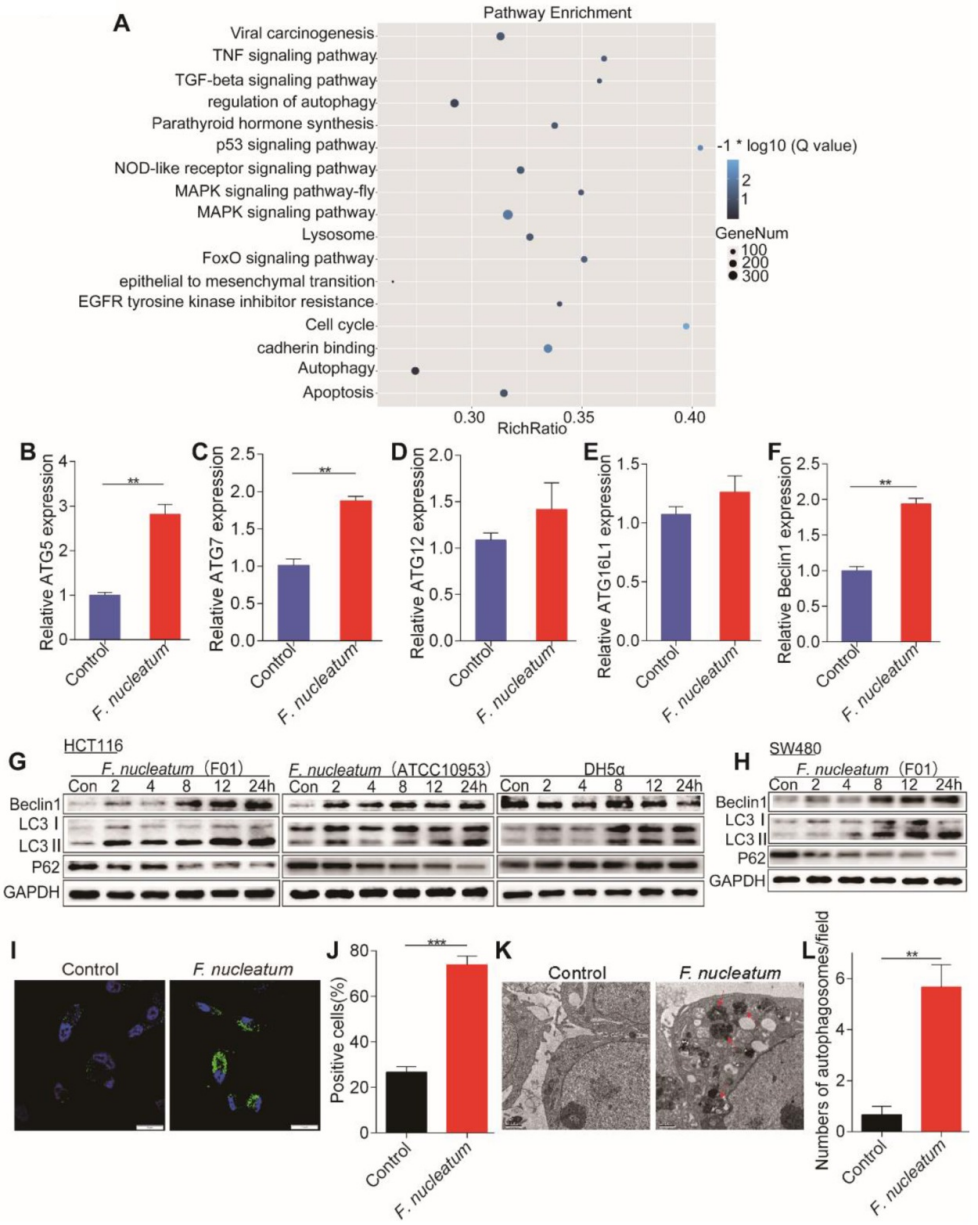
***F. nucleatum* Promotes Cancer Autophagy Activation *in vitro.***(A) ssGSEA was conducted to reveal the relationship between the abundance of *F. nucleatum* and the autophagy-related pathway activity in CRC cells. (B-F) Real-time PCR was performed in HCT116 cells cocultured with *F. nucleatum* (F01) or PBS (control) (*P < 0.05, **P < 0.01, and ***P < 0.001; unpaired Student's* t*-test; the bars indicate the SD of three experiments). (G-H) Western blot analysis was performed to detect autophagy element expression in HCT116 (G) and SW480 (H) cells cocultured with *F. nucleatum*, *E. coli* or PBS (control). (I-J) Immunofluorescence images of LC3-Ⅱ in HCT116 cells infected with or without *F. nucleatum* (F01; I). The positive cells were counted in five randomly chosen fields, and the data are presented as the means ± SDs (J; **P < 0.01; unpaired Student's *t*-test). (K-L) Representative electron micrographs of autophagosomes (red arrows) in HCT116 cells infected with or without *F. nucleatum* (F01) (K; scale bar, 0.5 µm). Quantification of cells containing autophagosomes (from K) in five randomly chosen fields, and the data are presented as the means ± SDs (L; *P < 0.05, and **P < 0.01; unpaired Student's *t*-test).

We hypothesized that *F. nucleatum* is biologically involved in the development of CRC metastasis. To test this hypothesis, we cocultured HCT116 cells with *F. nucleatum* (F01), performed RNA-seq analysis, and compared the gene expression profiles between HCT116 cells cocultured with or without *F. nucleatum* (F01). Infection with *F. nucleatum* (F01) downregulated the expression of 2966 genes and upregulated that of 1501 genes in HCT116 cells (adjusted *P* < 0.05). Single-sample gene set enrichment analysis (ssGSEA) revealed that the gene sets including the terms MAPK signaling pathway, lysosome and regulation of autophagy were enriched in HCT116 cells cocultured with *F. nucleatum* (F01) (adjusted* P* < 0.05; Figure 2A). Given the role of the autophagy pathway in metastasis 19, we reasoned that *F. nucleatum* (F01) may cause autophagy activation. Consistent with this hypothesis, *F. nucleatum* (F01) increased the mRNA expression of multiple autophagy-related signaling elements, including ATG5, ATG7 and Beclin1, in HCT116 cells (*P* < 0.01; Figure 2B-F). These data suggest that *F. nucleatum* (F01) may drive autophagy activation in HCT116 cells. To examine this possibility, we performed autophagy functional assays in HCT16 cells cocultured with *F. nucleatum* (F01). Western blot analysis showed time-dependent upregulation of LC3-Ⅱ and Beclin1 and downregulation of P62 in *F. nucleatum*-cocultured HCT116 cells (Figure 2G) and SW480 cells (Figure 2H) (*P* < 0.05). These effects were not found in HCT116 cells incubated with *E. coli* DH5a (Figure 2G). Furthermore, immunofluorescence showed that the percentage of cells containing LC3-Ⅱ puncta was increased after exposure to *F. nucleatum* (F01) for 24 h compared to that of control cells (*P* < 0.001; Figure 2I-J). Additionally, we evaluated autophagosomes in HCT116 cells cocultured with or without *F. nucleatum* (F01) for 24 h. Transmission electron microscopy showed an increase in the formation of autophagosomes in *F. nucleatum*-cocultured HCT116 cells (*P* < 0.01; Figure 2K-L). Collectively, our data indicate that *F. nucleatum* may activate the autophagy pathway in CRC cells.

### *F. nucleatum* Induces Cancer Metastasis via the Autophagy Pathway

**Figure 3:**
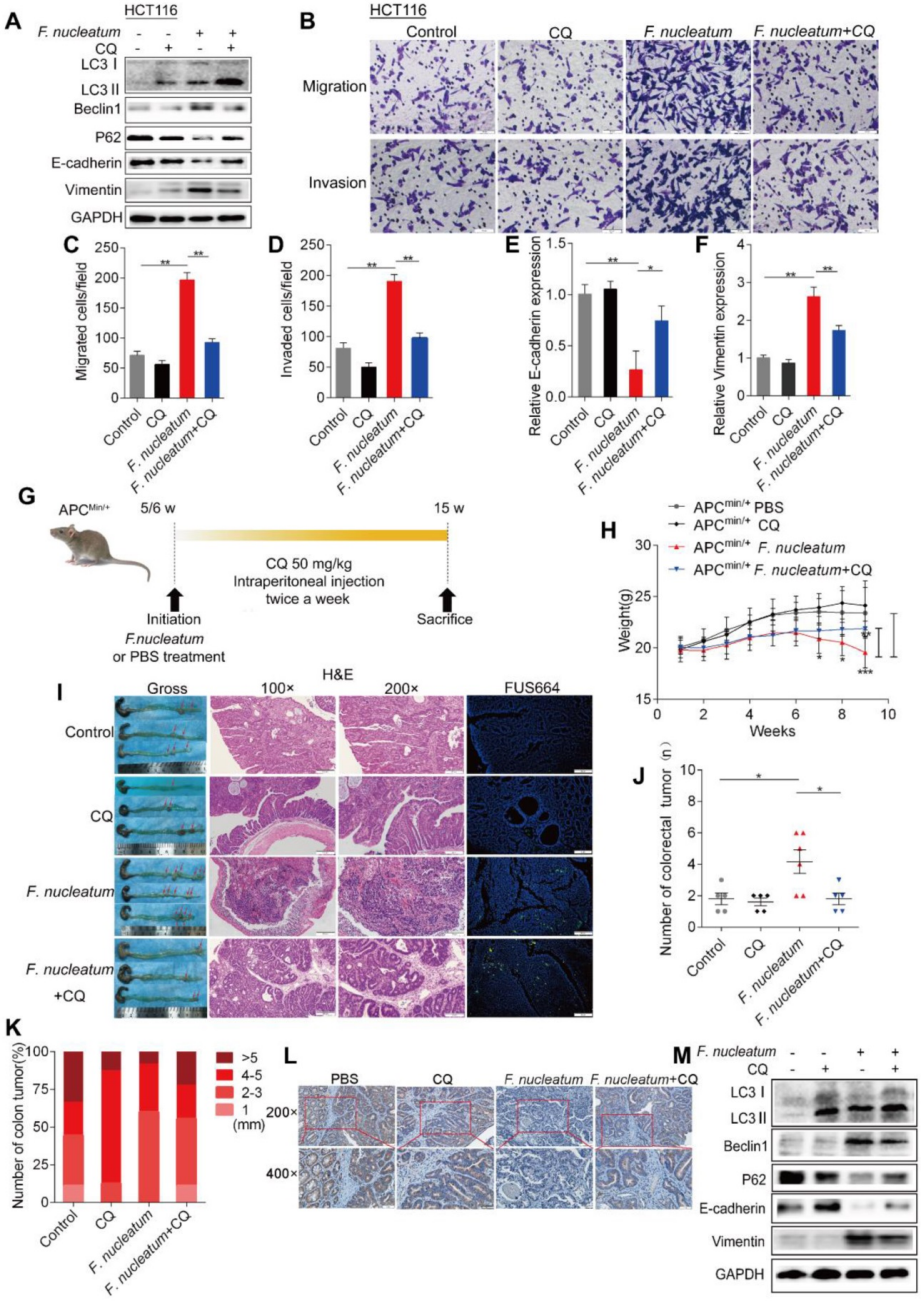
***F. nucleatum* Induces Cancer Metastasis via the Autophagy Pathway.** (A) Western blot analysis was performed in HCT116 cells cocultured with *F. nucleatum* (F01), the autophagy inhibitor CQ (20 µM) or PBS (control). (B-D) Transwell assays (B) were conducted with HCT116 cells cocultured with *F. nucleatum* (F01), CQ or PBS (control). The indicated migrated (C) and invaded (D) cells were quantified in five randomly selected fields, and the data are presented as the means ± SDs (**P < 0.01, and ***P < 0.001; unpaired Student's *t*-test). (E-F) The mRNA expression of E-cadherin (E) and Vimentin (F) was analyzed in HCT116 cells cocultured with *F. nucleatum* (F01), CQ or PBS (control) (*P < 0.05 and **P < 0.01; unpaired Student's *t*-test; the bars indicate the SD of three experiments) (G) Schematic of the experimental setup. (H-K) Representative colorectal tumors (red arrows) and H&E staining and FISH images of tumors in APC^Min/+^ mice treated with *F. nucleatum* (F01), CQ or PBS control (I). Statistical analysis of mouse weights (H; two-way ANOVA combined with Bonferroni's post hoc test), tumor numbers (J; nonparametric Mann-Whitney test) and tumor sizes (K) in the different groups (n = 5-6 mice/group; *P < 0.05, **P < 0.01, and ***P < 0.001; the error bars indicate the SDs). (L) E-cadherin expression in tumors from APC^Min/+^ mice treated with PBS (control), CQ, *F. nucleatum* (F01) or *F. nucleatum*(F01) + CQ was measured by immunohistochemical analysis. (M) Western blot analysis was performed with tissues from APC^Min/+^ mice in the different groups.

We next hypothesized that *F. nucleatum* induces cancer metastasis via autophagy. To initially test this hypothesis, we pretreated HCT116 cells with chloroquine (CQ; Sigma-Aldrich, C6628; 20 μM), an autophagic lysosomal inhibitor, for 3 h and subsequently treated them with *F. nucleatum* (F01) for 24 h. Pretreatment with CQ attenuated the *F. nucleatum*(F01)-mediated upregulation of Beclin1 and Vimentin expression and downregulation of P62 and E-cadherin expression (*P* < 0.05; Figure 3A). CQ increased E-cadherin expression and decreased Vimentin mRNA expression in *F. nucleatum* (F01) infected HCT116 cells (Figure 3E-F). Furthermore, we conducted transwell assays to investigate the effects of *F. nucleatum* (F01) on the motility of CRC cell lines. Compared with the corresponding control cells, HCT116 cells (Figure 3B) and SW480 cells (Figure S1B) cocultured with *F. nucleatum* (F01) displayed drastically enhanced cell migration and invasion (*P* < 0.05). However, *F. nucleatum* (F01) had no effect on HCT116 cells (Figure 3C-D) and SW480 cells (Figure S1C-D) pretreated with CQ. These data indicate that *F. nucleatum* may promote the metastasis of CRC cells via the autophagy pathway.

APC^Min/+^ mice carry adenomatous polyposis coli (APC) gene mutations, which predispose them to intestinal neoplasias. Here, we hypothesized that chronic *F. nucleatum* infection in APC^Min/+^ mice may induce a synergistic oncogenic effect and promote tumor metastasis via autophagy. To test our hypothesis, we administered *F. nucleatum* (F01), CQ or PBS to 5- to 6-week-old APC^Min/+^ mice (Figures 3G, S1E). Compared with control mice, APC^Min/+^ mice treated with *F. nucleatum* (F01) presented serious complications, including bloody stools (66.66% versus 20%; *P* = 0.242), splenomegaly (*P* = 0.015) and weight loss (*P* < 0.05; Figure 3H). CQ mildly attenuated *F. nucleatum*(F01)-mediated weight loss (*P* < 0.05, Figure 3H) but not the bloody stools (66.66% versus 60%; *P* > 0.05) or splenomegaly (*P* = 0.545). Notably, 8 weeks after *F. nucleatum* (F01) treatment, we found an increase in the numbers of colorectal tumors (Figure 3I;* P* = 0.026), and CQ treatment reversed this phenomenon (Figure 3J). *F. nucleatum* treatment increased the number of tumors in the whole intestine and small intestine (*P* < 0.05), but the numbers of tumors in these locations were reduced by CQ (*P* < 0.05; Figure S1F-H). There were no significant differences in the colorectal (Figure 3K) and intestinal tumor sizes (Figure S1I) in each group. These results suggest that *F. nucleatum* (F01) can observably stimulate tumor proliferation. In addition, compared with colorectal tumor tissues from the control and the *F. nucleatum* (F01) + CQ group, colorectal tumor tissues from *F. nucleatum*(F01)-treated mice showed more aggressive adenocarcinomas that invaded the muscular layer (Figure 3I). This finding indicates that *F. nucleatum* (F01) possibly increases tumor invasiveness. We further found that E-cadherin expression in CRC tissues from the *F. nucleatum* (F01) group was lower than that in tissues from the *F. nucleatum* (F01) + CQ group (Figure 3L). The Western blot analysis results showed that colorectal tumor tissues from *F. nucleatum* (F01)-treated mice expressed higher levels of LC3-Ⅱ, Beclin1 and Vimentin and lower levels of P62 and E-cadherin than those from control mice. CQ attenuated the *F. nucleatum* (F01)-mediated upregulation of Beclin1 and Vimentin and downregulation of P62 and E-cadherin (*P* < 0.05; Figure 3M). No metastasis was found in APC^Min/+^ mice, possibly due to the short modeling time. Despite this finding, we demonstrated that *F. nucleatum* may promote tumor metastasis via the autophagy pathway *in vivo* and *in vitro*.

### CARD3 Expression is Associated with *F. nucleatum*

**Figure 4:**
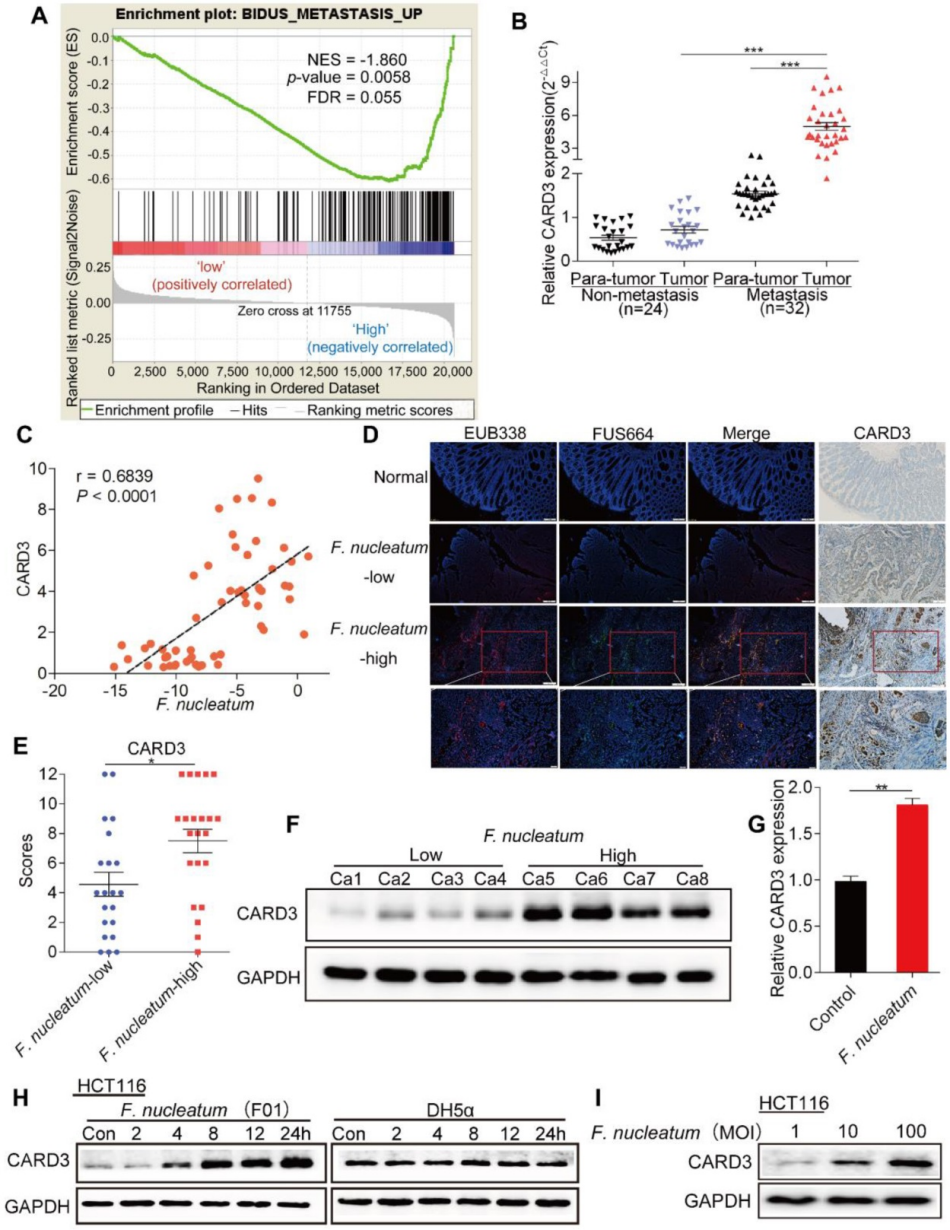
** CARD3 Expression is Associated with *F. nucleatum.***(A) GSEA for CARD3 expression with metastatic gene sets using the GSE21510 dataset (NES = -1.860, *P* = 0.0058, FDR = 0.055). (B-C) Statistical analysis of CARD3 mRNA expression in CRC tissues from patients with or without metastasis (B; ***P < 0.001; nonparametric Mann-Whitney test; the error bars indicate the SDs). Correlation analysis of the abundance of *F.nucleatum* and the expression of CARD3 in CRC tissues (C; r = 0.6839, *P* < 0.001; two-tailed, nonparametric Spearman correlation). (D-E) Representative images showing that the abundance of invasive *F. nucleatum* in CRC tissues is associated with CARD3 expression. 100× or 200× magnification (D). CARD3 protein levels in 21 *F. nucleatum-*low and 22 *F. nucleatum-*high CRC tissues from patients were measured by immunohistochemical analysis (E; *P < 0.05; unpaired Student's *t*-test; the error bars indicate the SDs). (F) Representative images of Western blots comparing the expression of CARD3 in *F. nucleatum-*low and *F. nucleatum-*high CRC tissues from patients. (G-I) Statistical analysis of CARD3 mRNA expression in HCT116 cells cocultured with *F. nucleatum* (F01, G). Western blot analysis was performed to measure CARD3 expression in HCT116 cells cocultured with *F. nucleatum* (F01) or *E. coli* in a time-dependent (H) or MOI-dependent (I) manner.

We performed gene set enrichment analysis (GSEA) to evaluate whether CRC metastasis is associated with CARD3 expression, and found that CARD3-enriched cases were associated with the metastasis gene signature (NES = -1.860461, *P* = 0.0058, FDR = 0.055; Figure 4A). We next analyzed the expression of CARD3 in 24 non-metastatic CRC tissues and 32 metastatic CRC tissues from the same patients referenced in Figure 1C. The real-time PCR results showed that CARD3 expression in tumor tissues from metastatic CRC patients was higher than that in tumor tissues from patients with non-metastatic CRC (*P* < 0.001, Figure 4B).

Combining these results with the results of the first part of the study, we hypothesized that CARD3 may be a target of *F. nucleatum* in CRC metastasis. To determine whether *F. nucleatum* affects CARD3 expression, we first conducted correlation analysis, and found that the amount of *F. nucleatum* (Figure 1C) was positively correlated with CARD3 mRNA expression (r = 0.6839, *P* < 0.0001, Figure 4C). We used FISH to visualize the abundance of *F. nucleatum* as well as the expression of CARD3 in CRC tissues and normal tissues. *F. nucleatum* invasion was found in CRC tissues, often accompanied by high levels of CARD3 (*P* < 0.05; Figure 4D-F). Additionally, the levels of CARD3 mRNA (Figure 4G) and protein (Figure 4H; *E. coli* DH5a as the control) in HCT116 cells were enhanced in response to *F. nucleatum* (F01) treatment in a time-dependent or MOI-dependent (Figure 2I) manner. These data support the hypothesis that CARD3 is probably a downstream target of *F. nucleatum*.

### CARD3 is Involved in *F. nucleatum*-Mediated Autophagy

**Figure 5:**
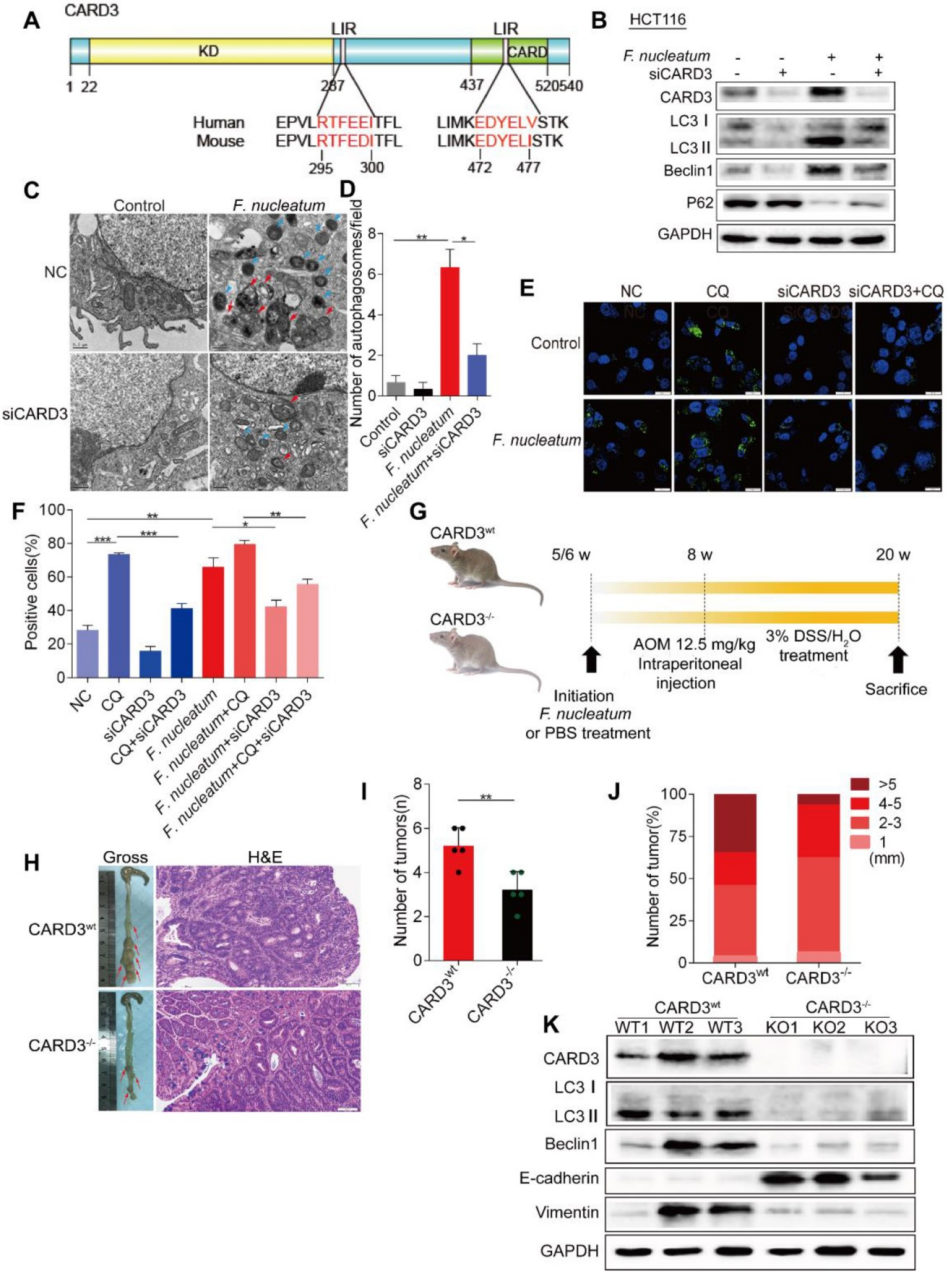
** CARD3 is Involved in *F. nucleatum*-Mediated Autophagy.** (A) Domain architecture of CARD3 and alignment of the LIR motifs (pink) between humans and rodents. (B) Western blot analysis was performed with HCT116 cells transfected with CARD3-targeting siRNA and cocultured with either PBS or* F. nucleatum* (F01). (C-D) Representative electron micrographs of autophagosomes (red arrows) in HCT116 cells infected with *F. nucleatum* (F01, blue arrows) or transfected with CARD3-targeting siRNA (C; scale bar, 0.5 µm). Quantification of cells containing autophagosomes (from C) was conducted with five random fields, and the data are presented as the means ± SDs (D; *P < 0.05, and **P < 0.01; unpaired Student's *t*-test). (E-F) Representative immunofluorescence images of LC3-Ⅱ in HCT116 cells (E; scale bar, 10 µm). The positive cells were counted in five random fields, and the data are presented as the means ± SDs (F; *P < 0.05, **P < 0.01, and ***P < 0.001; unpaired Student's *t*-test). (G) Schematic of the experimental setup. (H-J) Representative colorectal tumors (red arrows) and H&E staining of tumors from CARD3^wt^ and CARD3^-/-^ mice (I; 200× magnification). Statistical analysis of the tumor numbers (H) and sizes (J) in the different groups of mice (n = 5 mice/group; *P < 0.05; unpaired Student's *t*-test; the error bars indicate the SDs). (K) Western blot analysis was performed with CARD3^wt^ and CARD3^-/-^ mouse tissues.

CARD3 promotes autophagy and inflammatory signaling in response to bacterial infection 39. We used the iLIR web server (http://repeat.biol.ucy.ac.cy/iLIR/), a simple interface featuring protein sequence analysis tools 38, to predict whether the CARD3 protein can mediate autophagy. We found that CARD3 contains two LIR motifs for direct interaction with LC3 that are conserved in human and rodents (Figure 5A). Consistent with this finding, Western bloting showed that *F. nucleatum* (F01)-mediated autophagy activation was reduced in siCARD3 transfected HCT116 cells (*P* < 0.05; Figure 5B). *F. nucleatum*(F01)-induced autophagosomes were evaluated after CARD3 knockdown in HCT116 cells (Figure 5C). Transmission electron microscopy showed decrease accumulation of autophagosomes in *F. nucleatum*(F01) cocultured HCT116 cells transfected with siCARD3 (*P* < 0.05; Figure 5D). Knockdown of CARD3 decreased the *F. nucleatum* (F01)-induced increase in the percentage of cells containing LC3-Ⅱ puncta, and CQ further led to the accumulation of autophagosomes (*P* < 0.05; Figure 5E-F).

To further confirm our assumption that *F. nucleatum* promotes CRC progression through CARD3, we next employed CARD3 knockout (KO, CARD3^-/-^) mice. AOM is a recognized procarcinogen with organotropism for the colon, and AOM-induced tumorigenesis is significantly enhanced by DSS. As shown in Figure 5G, both CARD3 wild-type (CARD3^wt^) and CARD3^-/-^ mice were initially administered *F. nucleatum* (F01) and then subjected to AOM/DSS treatment. The colorectum in CARD3^-/-^ mice had tumors with higher histological grades than those in CARD3^wt^ mice (Figure 5I) and had fewer tumors (*P* < 0.01; Figure 5H) with smaller sizes (6.25% versus 34.62% for > 5 mm tumors; Figure 5J) than the colorectum in CARD3^wt^ mice. Furthermore, Western blot analysis showed that colorectal tumor tissues from CARD3^-/-^ mice expressed lower levels of LC3-II, Beclin1 and Vimentin and higher levels of E-cadherin than tumor tissues from CARD3^wt^ mice (*P* < 0.05; Figure 5K). Thus, these data indicate that *F. nucleatum* may activate cancer-associated autophagy via upregulation of CARD3 and that CARD3 knockdown/knockout can inhibit the oncogenic function of *F. nucleatum* during colorectal carcinogenesis.

### CARD3 Regulates *F.nucleatum*-Mediated Metastasis through Autophagy

**Figure 6:**
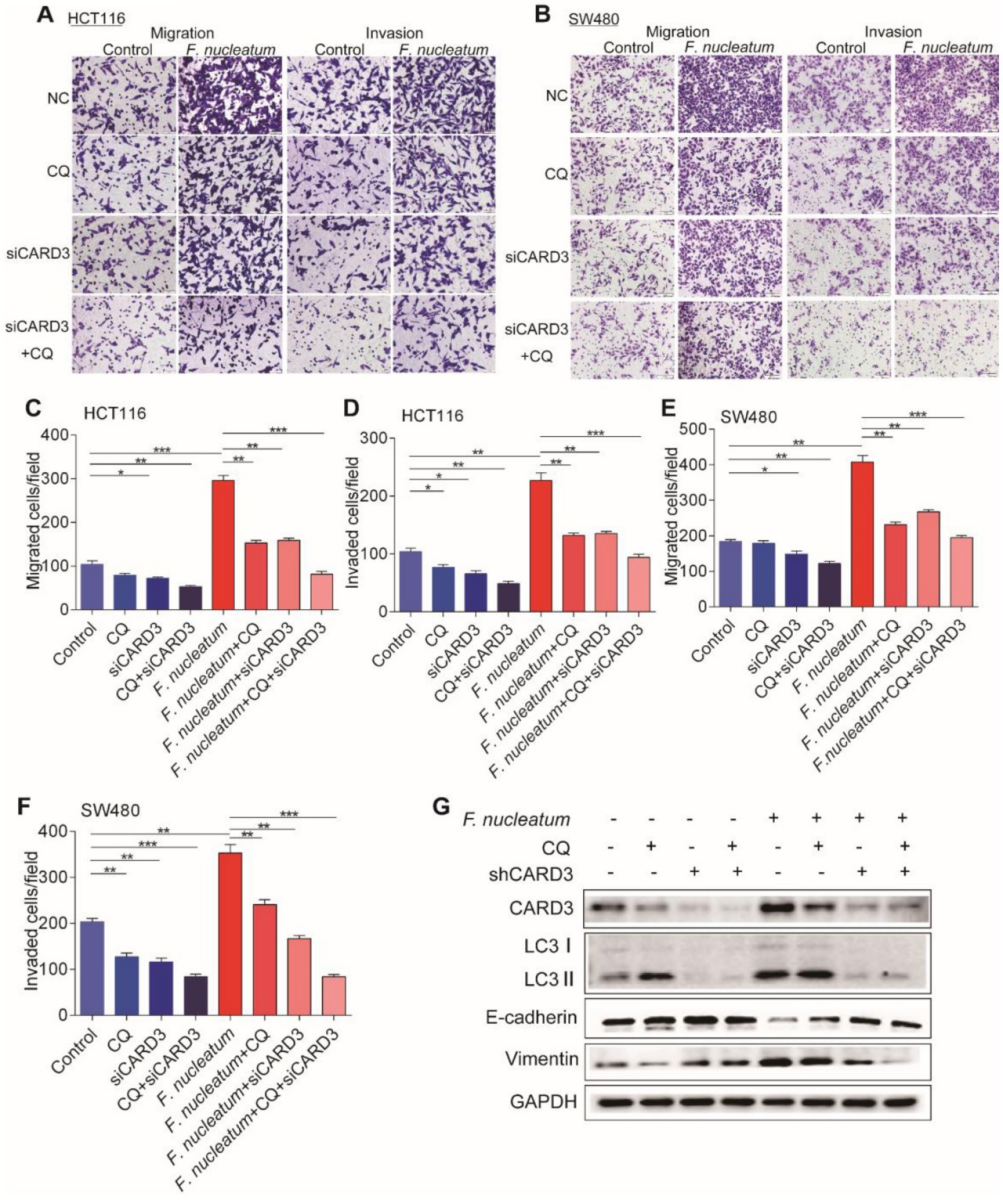
** CARD3 is Involved in *F. nucleatum*-Mediated Autophagy *in vitro.***(A-F) Transwell assays were conducted with HCT116 cells (A) and SW480 cells (B) treated with *F. nucleatum* (F01), CQ or siCARD3. The indicated migrated and invaded cells were quantified in five random fields, and the data are presented as the means ± SDs (C-F; *P < 0.05, **P < 0.01, and ***P < 0.001; unpaired Student's *t*-test). (G) Western blot analysis was performed with HCT116 cells treated with *F. nucleatum* (F01), CQ or siCARD3.

The increase of CARD3 expression in *F. nucleatum*-induced autophagy led us to hypothesize that CARD3 may regulate *F. nucleatum*-mediated metastasis by activating the autophagy pathway. To test this hypothesis, we performed CARD3-targeting siRNA transfection or CQ treatment in CRC cells cocultured with *F. nucleatum* (F01). siCARD3, CQ or CQ + siCARD3 reduced *F. nucleatum* (F01)-induced invasion and migration of HCT116 cells (*P* < 0.05; Figure 6A, C-D) and SW480 cells (*P* < 0.05; Figure 6B, E-F). Moreover, Western blotting showed that *F. nucleatum* (F01) induced upregulation of CARD3, LC3-Ⅱ and Vimentin and downregulation of E-cadherin and that these effects were blocked in HCT116 cells by transfection of CARD3-targeting siRNA or pretreatment with CQ (*P* < 0.05; Figure 6G). Thus, CARD3 knockdown may protect CRC cells from *F. nucleatum* (F01)-induced invasion and metastasis via the autophagy pathway.

Blood-borne *F. nucleatum* can localize to CRC 40. To determine the length of time that the circulatory system can support *F. nucleatum* infection, we injected 5×10^6^ to 1×10^7^ *F. nucleatum* (F01) into wild-type BALB/cJ mice via the tail vein and found that mice can withstand blood-borne infection with *F. nucleatum* (F01) for an extended duration. Thus, to investigate whether the ability of CQ treatment or CARD3 knockdown to inhibit *F. nucleatum*-induced cancer cell migration and invasion *in vitro* translates into decreased metastasis *in vivo*, we used an experimental lung metastasis model with BALB/cJ mice.

**Figure 7:**
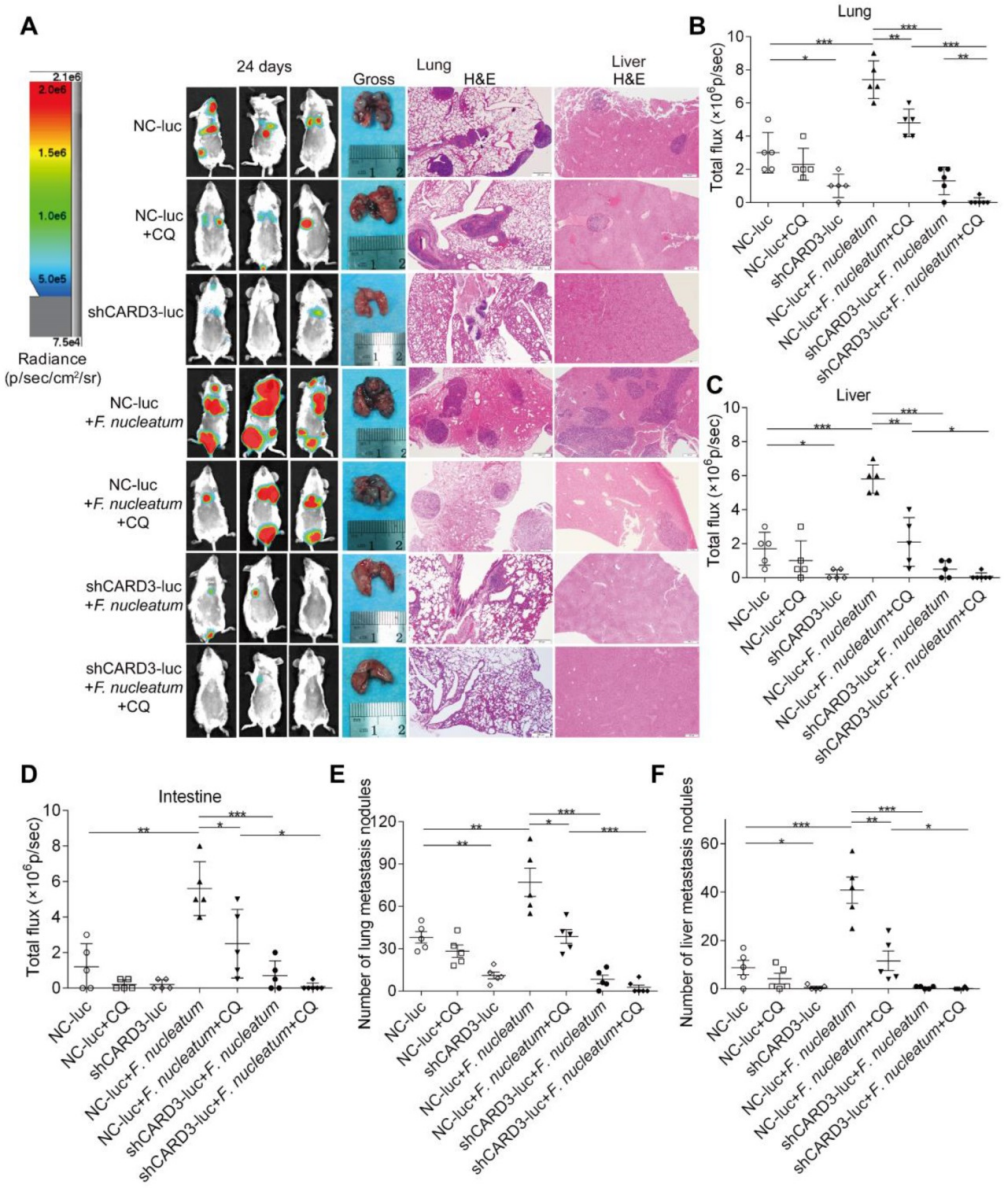
** CARD3 is Involved in *F. nucleatum*-Mediated Autophagy *in vivo.***(A-D) Bioluminescence imaging (BLI) was used to monitor metastases (A) in the lungs (B), livers (C), and intestines (D) of wild-type BALB/cJ mice (n = 5-6 mice/group). Representative gross lungs and H&E-stained lung and liver sections from mice (A). The data are presented as the means ± SDs (*P < 0.05, **P < 0.01, and ***P < 0.001; Mann-Whitney test). (E-F) The metastatic foci in lungs (E) and livers (F) were counted. The data are presented as the means ± SDs. (*P < 0.05, **P < 0.01, and ***P < 0.001; Mann-Whitney test).

The following seven experimental groups were tested: (i) NC-luc, (ii) NC-luc + CQ, (iii) shCARD3-luc, (iv) NC-luc + *F. nucleatum*, (v) NC-luc + *F. nucleatum* + CQ, (ⅵ) shCARD3-luc + *F. nucleatum*, and (ⅶ) shCARD3-luc + *F. nucleatum* + CQ. Tumor growth was examined by luciferase imaging. The total number of visible surface lung and liver metastases was counted (regardless of their sizes), and lung and liver tissue sections were further analyzed by H&E staining (Figure 7A). Mice injected with *F. nucleatum* (F01) exhibited increased formation of metastatic nodules in the lungs (*P* < 0.001; Figure 7B), livers (*P* < 0.001; Figure 7C) and colons (*P* < 0.01; Figure 7D) compared with mice injected with PBS. Treatment with CQ mildly reduced the invasive activity of CT26 cells and formation of metastases induced by *F. nucleatum*. Both CARD3 disruption and combination treatment significantly reduced the ability of *F. nucleatum*-mediated CT26 cells to form metastases in the lungs (*P* < 0.001; Figure 7E) and livers (*P* < 0.05; Figure 7F) following injection of these cells into mice. These data indicate that genetic disruption of CARD3 may reduce the *F. nucleatum*-mediated invasive activity of CRC cells and their ability to form metastases following injection into mice.

In summary, we conclude that *F. nucleatum* exacerbates CRC via the upregulation of CARD3 expression, subsequently resulting in autophagy activation and promoting* F. nucleatum*-mediated metastasis.

## Discussion

Most patients with CRC die from metastasis 41,42. Thus, understanding the mechanisms of metastasis in CRC is essential for optimizing current therapeutic strategies.

Metagenomic and transcriptomic analyses have revealed that gut microbiota, especially *F. nucleatum*, are involved in CRC development 9,10. Studies in diverse experimental models have suggested a protumorigenic role for *F. nucleatum*. Infection of CRC cells with *F. nucleatum* 14,36, feeding mice with *F. nucleatum* 11,43, and generation of xenografts derived from *F. nucleatum*-infected colorectal cancer cells 11 were found to potentiate tumor cell growth. The suggested mechanisms range from the enhancement of tumor cell adhesion, proliferation and invasion 11,14,40 to the modulation of the host immune response 35,43-45 to the activation of cancer-associated pathways 11,14, to promotion of recurrence and chemoresistance 13. However, the potential role of *F. nucleatum* in CRC metastasis is poorly understood. Our high-throughput sequencing and functional studies elucidated that *F. nucleatum* abundance is increased in metastatic CRC tissues and is correlated with CRC metastasis. Interestingly, a recent study provided novel insights that *Fusobacterium* can be detected in liver metastases and may migrate with CRC cells to metastatic sites 15. Consistent with these findings, we showed that *F. nucleatum* was present in metastases. Furthermore, our results revealed the first evidence of crosstalk between *F. nucleatum* and EMT *in vitro* and *in vivo*. Through a combination of genomic, biological, *in vivo* models and clinical studies, we demonstrated that a high abundance of* F. nucleatum* is present in metastatic CRC tissues, and that *F. nucleatum* may promote CRC metastasis.

The interaction between a host polysaccharide, Gal-GalNAc with fusobacterial lectin Fap2, facilitates *F. nucleatum* enrichment in CRC tissues 40. The *F. nucleatum* adhesin FadA may bind to the E-cadherin protein and promote colorectal carcinogenesis 14. *F. nucleatum* mediates chemoresistance by selectively targeting specific miRNAs and autophagy elements 13. However, it is unknown how *F. nucleatum* mediates metastasis in CRC. Our transcriptomics studies also demonstrated that *F. nucleatum* (F01), which was isolated from the colon tissues of CRC patients, may regulate autophagy. We found that *F. nucleatum* (F01) induced LC3-Ⅱ expression, autophagic flux, and autophagosome synthesis and stimulated the expression of autophagy-related proteins or mRNAs, such as P62, Beclin1, ATG5 and ATG7, in CRC cells. Biochemical autophagy inhibition reduced *F. nucleatum*(F01)-induced EMT *in vitro* and* in vivo*. Thus, we conclude that autophagy possibly contributes to *F. nucleatum*-mediated CRC metastasis.

Furthermore, we analyzed the mechanisms by which *F. nucleatum* mediates the activation of the autophagy pathway. It has been reported that pathogenic bacteria induce autophagosome formation and inflammatory responses in epithelial cells in a CARD3-dependent manner 22. Previous studies have shown that CARD3 is a prometastatic kinase 20,24. In this study, our bioinformatic analyses showed that the CARD3 protein contains two LIR motifs for direct interaction with LC3. In addition, we demonstrated that CARD3 expression was higher in patients with metastasis than in patients without metastasis and that *F. nucleatum* abundance was positively correlated with CARD3 expression. Knockdown of CARD3 expression decreased *F. nucleatum* (F01)-induced migration, invasion, autophagosome formation, autophagy-related protein expression, and metastasis *in vitro* or *in vivo*. Thus, we identified a new mechanism by which *F. nucleatum* activates autophagy to promote CRC metastasis by specifically targeting CARD3. However, we did not dissect the mechanisms by which CARD3 mediates the activation of the autophagy pathway. Studies are needed to determine how *F. nucleatum* induces autophagic flux via CARD3 and to precisely understand how *F. nucleatum* is related to CARD3 upregulation.

A previous study showed that when mice were inoculated with 1×10^6^ to 1×10^7^ *F. nucleatum* via tail vein injection, blood-borne *F. nucleatum* localized to CRC 40. Consistently, we first established an experimental lung metastasis model using injections of a mixture of CT26 cells and *F. nucleatum* (F01) and found that mice can withstand blood-borne infection with *F. nucleatum* (F01) for an extended duration and that *F. nucleatum* (F01) can promote CRC cell migration *in vivo*. However, this model did not reveal whether colorectal cancer cells metastasize together with *F. nucleatum* or identify the domains via which *F. nucleatum* binds to CRC cells. Binding of the Fap2 protein of *F. nucleatum* to the human inhibitory receptor TIGIT can protect tumors from immune cell attack 35. In the future, it will be interesting to determine the exact *F. nucleatum* domain (s) involved in cancer metastasis and to investigate whether blood-borne *F. nucleatum* and CRC cell migration are associated with inhibition of tumor immunity.

From a clinical viewpoint, our data may be relevant to the management of CRC patients. As the amount of *F. nucleatum* is associated with the risk of CRC metastasis, measuring *F. nucleatum* abundance may be an effective approach to predict metastasis in patients. Furthermore, our data raise an important clinical question: should CRC patients with a high amount of *F. nucleatum* be treated with conventional chemotherapy in combination with anti-*F. nucleatum* treatment and/or an autophagy inhibitor? Our findings support the hypothesis that targeting *F. nucleatum*, host epithelial CARD3 expression and/or autophagy may provide a means to block* F. nucleatum*-induced CRC metastasis and create diagnostic opportunities. Thus, measuring* F. nucleatum* abundance and its associated pathway and differentially managing patients with different levels of *F. nucleatum* is important.

In summary, our current findings provide critical insights into the molecular mechanisms underlying the regulation of CARD3 by *F. nucleatum* in CRC metastasis. In addition, the clinical results presented here highlight both *F. nucleatum* and CARD3 as risk factors for metastasis in colorectal cancer patients. Our data provide previously unrecognized and novel evidence for a prometastatic role of *F. nucleatum* in CRC, offering new opportunities for targeting microbiota and gene alterations for the prevention and treatment of colorectal cancer metastasis.

## Figures and Tables

**Table 1:** Clinicopathologic characteristics in *F. nucleatum*-low vs. *F. nucleatum*-high colorectal cancers

Characteristics	*F. nucleatum*.		*p-*value^a^
	Low (n=33)	High (n=61)	
**Gender**			0.551
Female	16	29	
Male	17	32	
**Age**	59.94	61.20	0.460
(Range )	25-81	23-86	
**Location**			0.031*
Proximal	9	31	
Distal	24	30	
**Tumor size**			0.423
d<4cm	8	19	0.002*
4≤d<8cm	22	40	
d≥8cm	3	2	
**AJCC stage**			
Ⅰ-Ⅱ	18	14	
Ⅲ-Ⅳ	15	47	
**Histologic grade**			0.065
Well differentiated	6	3	
Moderately differentiated	24	46	
Poorly differentiated	3	12	
**Pathological type**			0.709
eminence type	18	29	
ulcer type	11	21	
Infiltrating type	4	11	
**Depth of invasion**			0.023*
T1	2	2	
T2	10	5	
T3	20	47	
T4	1	7	
**Lymph node metastasis**			0.005*
N0	19	16	
N1	9	19	
N2	5	26	
**Distant metastasis**			0.037*
M0	32	50	
M1	1	11	

^a^ Chi-square test or Fisher's exact test, *P < 0.05; AJCC, American Joint Committee On Cancer.

## Abbreviations

CRC: colorectal cancer
*F. nucleatum*: *Fusobacterium nucleatum*
*E. coli*: *Escherichia coli*
CARD3: caspase activation and recruitment domain 3
NF-κB: nuclear factor-kappa B
EMT: epithelial-mesenchymal transition
CQ: chloroquine
AOM: azoxymethane
DSS: dextran sodium sulfate
FISH: fluorescence *in situ* hybridization
IHC: immunohistochemistry
WB: western blotting
MOI: multiplicity of infection
