# *Fusobacterium nucleatum* Promotes Metastasis in Colorectal Cancer by Activating Autophagy Signaling via the Upregulation of CARD3 Expression: Erratum

**DOI:** 10.7150/thno.69114

**Published:** 2022-01-01

**Authors:** Yongyu Chen, Yan Chen, Jixiang Zhang, Pan Cao, Wenhao Su, Yunchao Deng, Na Zhan, Xiangsheng Fu, Yun Huang, Weiguo Dong

**Affiliations:** 1 Department of Gastroenterology, Renmin Hospital of Wuhan University, Wuhan, Hubei Province, China.; 2 Key Laboratory of Hubei Province for Digestive System Disease, Wuhan, Hubei Province, China.; 3 Central Laboratory, Renmin Hospital of Wuhan University, Wuhan, Hubei Province, China.; 4 Department of Gastroenterology, The Affiliated Hospitalof North Sichuan Medical College, Road Wenhua 63#, Region Shunqing, Nanchong City 637000, China.; 5 Center for Epigenetics & Disease Prevention, Institute of Biosciences and Technology, Texas A&M University, Houston, TX77030, USA.

The authors regret that the original version of our paper 1 unfortunately contained two inappropriate representative images of WB. Noting that WB images (the Vimentin of ATCC10953 and the CARD3 of F01) in the first submitted version were correct. For presenting high resolution images according to one of the reviewer's suggestion, we assembled inappropriate images in our revised manuscript (Figure 1I and Figure 4H). We regret that at the time of figure assembly, we choose representative images by mistake. Besides, we apologize for the original version of our paper unfortunately contained some inappropriate representative images of transwell. We mixed up some of them, which caused some confusion in Figure 3B, Figure 6AB and Supplementary Figure 1B. Although we have checked the paper before submission, we have not been careful enough to find out the problems in time. We apologize for our careless at the time of figure assembly. We confirm that it would not affect any results and conclusions of the paper. The authors apologize for any inconvenience that this error may have caused.

## Figures and Tables

**Figure 1:**
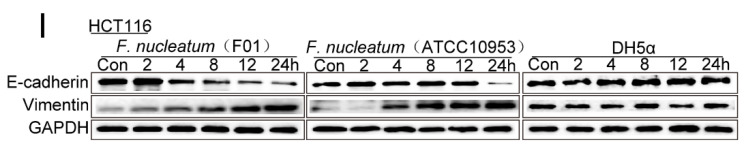
Corrected image for original Figure 1I.

**Figure 4:**
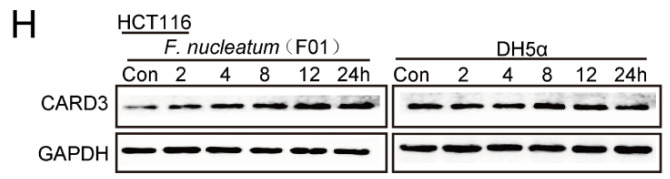
Corrected image for original Figure 4H.

**Figure 3:**
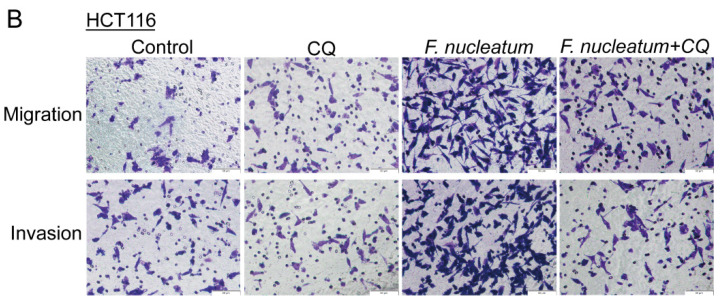
Corrected image for original Figure 3B.

**Figure 6:**
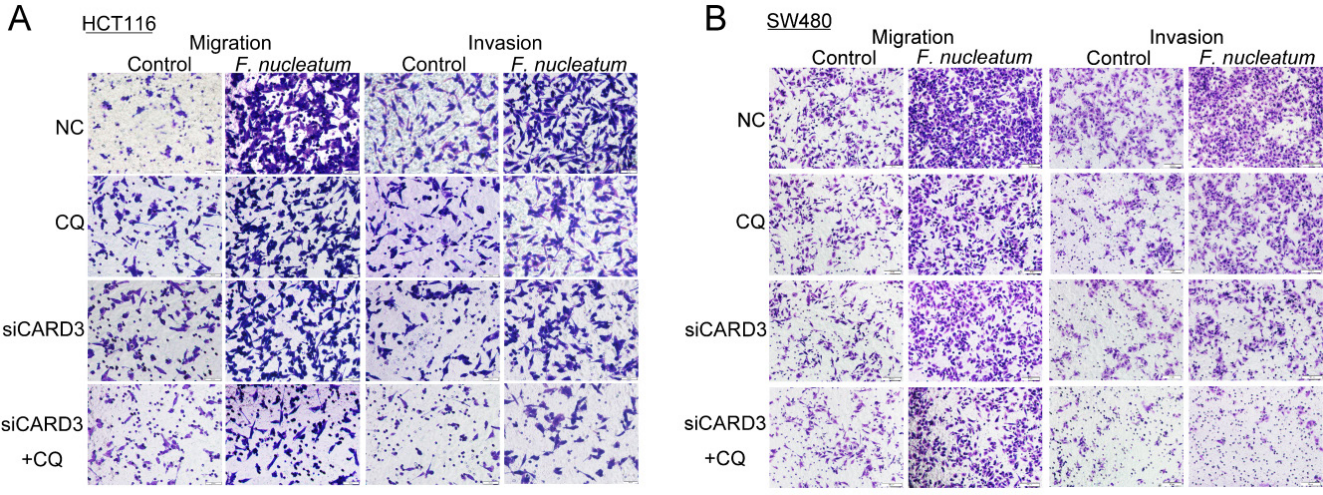
Corrected image for original Figure 6AB.

**Figure A:**
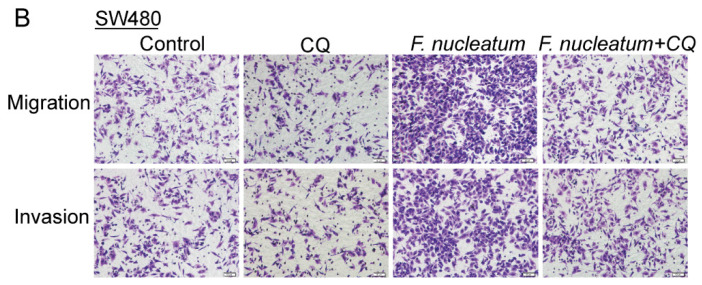
Corrected image for original Figure S1B.

## References

[1] Chen Y, Chen Y, Zhang J, Cao P, Su W, Deng Y, Zhan N, Fu X, Huang Y, Dong W (2020). *Fusobacterium nucleatum* Promotes Metastasis in Colorectal Cancer by Activating Autophagy Signaling via the Upregulation of CARD3 Expression. *Theranostics*.

