# Supplementary material for: Fusobacterium nucleatum Promotes Metastasis in Colorectal Cancer by Activating Autophagy Signaling via the Upregulation of CARD3 Expression
Source: Theranostics. 2020 Jan 1;10(1):323–39. doi: 10.7150/thno.38870 (PMC6929621; doi:10.7150/thno.38870)
Supplement: Supplementary file 1 — Supplementary figures and tables. [file thnov10p0323s1.pdf]

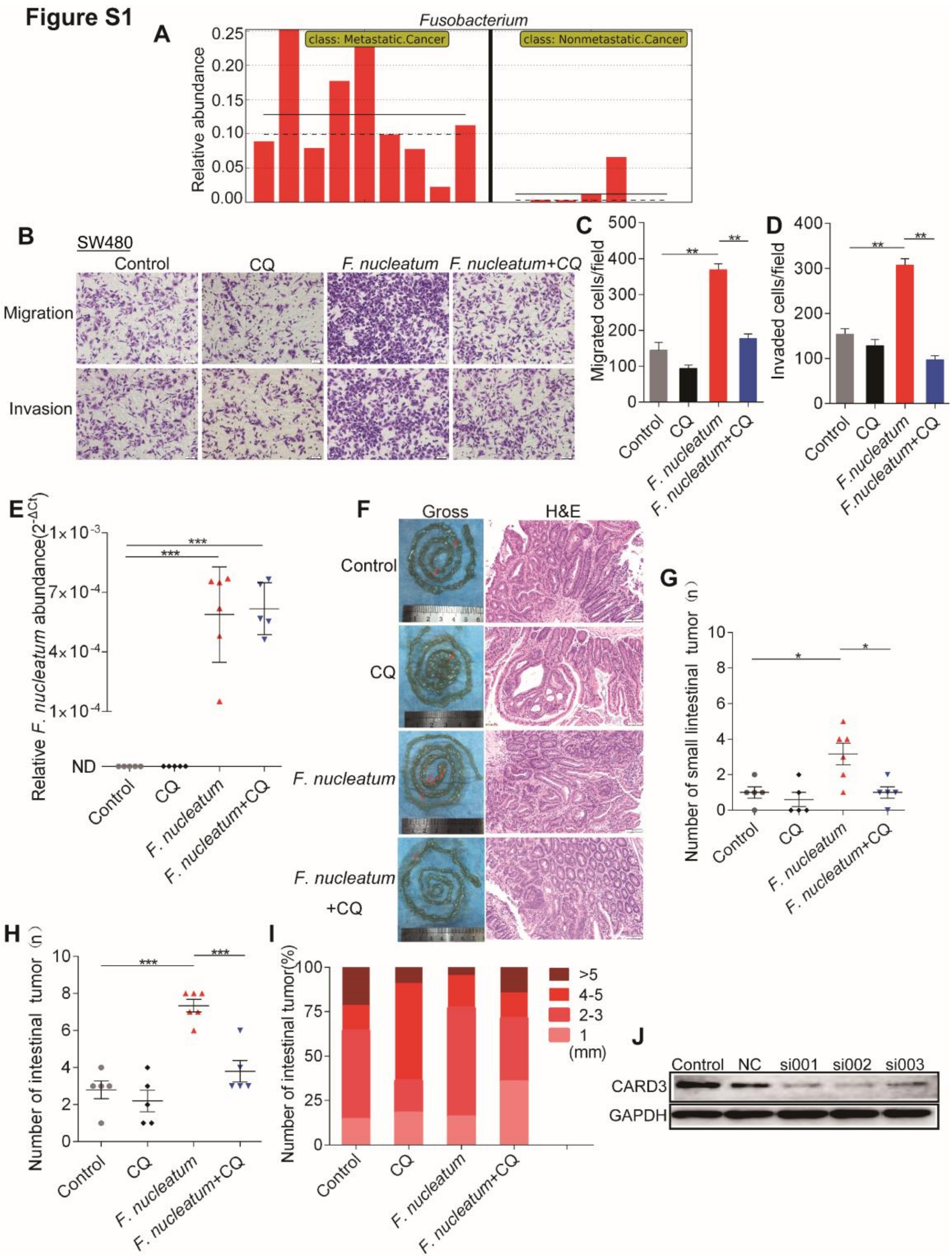

Figure S1. *F. nucleatum* Induces Cancer Metastasis via the Autophagy Pathway

(A) 16S rDNA analysis of the relative abundance of *Fusobacterium* in CRC tissues from patients without metastasis (n = 7) and with metastasis

(n = 9).

(B-D) Transwell assays were conducted to examine the migratory and invasive abilities of SW480 cells cocultured with *F. nucleatum* (F01), CQ or PBS control. The indicated migrated and invaded cells were quantified in five randomly selected fields, and the data are presented as the means ± SDs (***P* < 0.01; unpaired Student's *t*-test).

(E) Relative fusobacterial gDNA abundance ($2^{-\Delta Ct}$) in tumor tissues from $APC^{Min/+}$ mice with *F. nucleatum* (F01), CQ or PBS. (n = 5-6 mice/group; the error bars indicate the means ± SDs; ****P* < 0.001, Mann-Whitney U test).

(F-I) Representative data for small intestinal tumors (red arrows) and HE staining of tumor tissues from the indicated groups of $APC^{Min/+}$ mice (F). Statistical analysis of the small intestinal tumor numbers (G), whole intestinal tumor numbers (H) and tumor sizes (I) in different groups of mice (n = 5-6 mice/group; **P* < 0.05, and ****P* < 0.001; nonparametric Mann-Whitney test).

(J) Western blot analysis was performed with HCT116 cells transfected with non-targeting (NC) or CARD3-targeting (si001, si002, si003) siRNA.

Supplementary Table 1

Primers		Sequence5'-3'	Purpose
F. nucleatum	Forward	5'- CAACCATTACTTTAACTCTACCATGTTCA -3'	qPCR
	Reverse	5'- GTTGACTTTACAGAAGGAGATTATGTAAAAATC -3'	
PGT	Forward	5'- ATCCCCAAAGCACCTGGTTT -3'	qPCR
	Reverse	5'- AGAGGCCAAGATAGTCCTGGTAA -3'	
H-GAPDH	Forward	5'- CATCATCCCTGCCTCTACTGG -3'	qPCR
	Reverse	5'- GTGGGTGTCGCTGTTGAAGTC -3'	
H-E-cadherin	Forward	5'- TTCTTCGGAGGAGAGCGG -3'	qPCR
	Reverse	5'- CAATTTCATCGGGATTGGC -3'	
H-CARD3	Forward	5'- GCTGCATTCTGTGATCACAAGAC -3'	qPCR
	Reverse	5'- GACAGAAGGGCATCTAGCGAC -3'	
H-Vimentin	Forward	5'- AGGCAAAGCAGGAGTCCACT -3'	qPCR
	Reverse	5'- CGTTCCAGGGACTCATTGGT -3'	
H-ATG5	Forward	5'- TGTTTATTCGTCGGTTCATTTTG -3'	qPCR
	Reverse	5'- CAGCTTAGTGTTCCCTGCATTC -3'	
H-ATG12	Forward	5'- CTACTTCAATTGCTGCTGGAGG -3'	qPCR
	Reverse	5'- GCCAGCAGGTTCCTCTGTTC -3'	
H-ATG16L1	Forward	5'- GCTAAGTTCCTGCTGGACAATG -3'	qPCR
	Reverse	5'- GGCAGTAATCTTTCCCAACAGC -3'	
H-ATG7	Forward	5'- TTCCTCCTCTTGACATTTGCAG -3'	qPCR
	Reverse	5'- TATCTTCGTCCTTTGACCTTGG -3'	
H-beclin1	Forward	5'- GGCACAATCAATAACTTCAGGC -3'	qPCR

Reverse	5’- GGCAGCTCCTTAGATTTGTCTG -3’
